# A halogen-free synthesis of gold nanoparticles using gold(III) oxide

**DOI:** 10.1007/s11051-016-3576-x

**Published:** 2016-08-29

**Authors:** Volodymyr Sashuk, Konrad Rogaczewski

**Affiliations:** Institute of Physical Chemistry, Polish Academy of Sciences, 01-224 Warsaw, Poland

**Keywords:** Gold nanoparticles, Gold(III) oxide, Oleylamine, Bimetallic nanoparticles

## Abstract

Gold nanoparticles are one of the most used nanomaterials. They are usually synthesized by the reduction of gold(III) chloride. However, the presence of halide ions in the reaction mixture is not always welcome. In some cases, these ions have detrimental influence on the morphology and structure of resulting nanoparticles. Here, we present a simple and halogen-free procedure to prepare gold nanoparticles by reduction of gold(III) oxide in neat oleylamine. The method provides the particles with an average size below 10 nm and dispersity of tens of percent. The process of nanoparticle formation was monitored using UV–Vis spectroscopy. The structure and chemical composition of the nanoparticles was determined by SEM, XPS and EDX. We also proposed the mechanism of reduction of gold(III) oxide based on MS, IR and NMR data. Importantly, the synthetic protocol is general and applicable for the preparation of other coinage metal nanoparticles from the corresponding metal oxides. For instance, we demonstrated that the absence of halogen enables efficient alloying of metals when preparing gold–silver bimetallic nanoparticles.

**Electronic supplementary material:**

The online version of this article (doi:10.1007/s11051-016-3576-x) contains supplementary material, which is available to authorized users.

## Introduction

Gold nanoparticles (AuNPs)—due to chemical robustness, easy preparation and functionalization—are often used as a model system for studying nanoscale phenomena (Daniel and Astruc 2004; Hakkinen 2012; Kalsin et al. 2006; Nealon et al. 2012; Sashuk 2012; Sashuk et al. 2012, 2013; Teulle et al. 2015; Wells et al. 2015; Zhao et al. 2016). Moreover, the pristine chemical and physical properties of nanoscopic gold provide additional appeal for applications such as catalysis or sensing (Giljohann et al. 2010; Jans and Huo 2012; Kale et al. 2014; Mikami et al. 2013; Saha et al. 2012; Stratakis and Garcia 2012; Yeh et al. 2012). AuNPs are routinely synthesized by reducing gold halides, typically gold(III) chloride (Bhargava et al. 2005; Brust et al. 1994; Dozol et al. 2013; Jana et al. 2001; Jana and Peng 2003; Kimling et al. 2006; Lee et al. 2010; Leff et al. 1996; Martin et al. 2010; Perrault and Chan 2009; Zhao et al. 2013). The role of halogen in this process is generally overlooked since the size and shape—two basic parameters of the NP—can be controlled by reductants and ligands employed (Scarabelli et al. 2014; Zhang et al. 2014). Though, the halide ions present in a solution can affect the morphology and surface chemistry of NPs (Li et al. 2013; Rai et al. 2006; Singh et al. 2007). The presence of halides in a reaction mixture can also influence the final composition of NPs, e.g. Au–Ag alloys (Rajendra et al. 2015). Hence, we wondered whether gold oxide could be an alternative to gold chloride. The both gold precursors are commercially available on comparable prices that were an additional reason to pursue the research. Meisel and co-workers have recently shown that gold(III) oxide undergoes decomposition to gold colloids upon reduction by molecular hydrogen (Merga et al. 2010). The method, however, requires a special set-up by virtue of the risk of gas explosion that restricts significantly its practical utility. Also, the as-prepared NPs are quite large (20–100 nm) displaying a moderate degree of monodispersity. From application point of view, the smaller nanoparticles with narrow size distribution would be more desirable because of, e.g. more pronounced intrinsic properties, better resistance against aggregation and suitability for studying self-assembly processes.

Herein, we present a simple and convenient method to obtain gold nanoparticles by reduction of gold(III) oxide with oleylamine. Remarkably, the amine (Mourdikoudis and Liz-Marzán 2013; Yu et al. 2014) acts as all-in-one reagent with functions of reductant, ligand and reaction medium. The reaction yields NPs with a small mean size (5–9 nm) and reasonable polydispersity (<1.5). Thus, the method offers a specific size range not accessible from classical oleylamine reduction route based on gold(III) chloride. For instance, the NPs obtained under solution processing conditions were usually above 9 nm (Aslam et al. 2004; Fanizza et al. 2013; Hiramatsu and Osterloh 2004; Lakshminarayana and Qing-Hua 2009; Shen et al. 2008). On other hand, mechanochemical synthesis performed in bulk afforded ultra-small particles with diameters between 1 and 5 nm (Rak et al. 2014). The use of gold(III) oxide affects not only the size of NPs but also enables to preserve the initial metal ratio when preparing Au–Ag alloy NPs. In contrast, the alloys obtained from gold(III) chloride are characterized by reduced silver content owing to solubility issues.

## Experimental

### Materials

All chemicals were purchased from commercial suppliers and used without further purification: Au_2_O_3_ (99 %, ABCR), Ag_2_O (99 %, Sigma-Aldrich), HAuCl_4_·3H_2_O (Sigma-Aldrich), AgNO_3_ (99 %, Alfa Aesar), oleylamine (technical grade, 70 %, Sigma), octylamine (for synthesis, Merck), dodecylamine (98 %, Aldrich), hexadecylamine (98 %, Aldrich), octadecylamine (98 %, Alfa Aesar), triphenylphosphine (95 %, Fluka) and 1-undecanthiol (98 %, Aldrich). The solvents were of analytical grade quality and degassed by freeze-pomp-thaw technique prior to use: toluene, chlorobenzene, chloroform (ChemPur), decane, tetradecane (Aldrich) and 1,2-dichlorobenzene (ROTH). Silicon wafers were received from ITME (Warsaw). TEM grids were purchased from Ted Pella Inc.

### Instrumentation

NMR spectra were recorded on Bruker (400 MHz) instrument. GC–MS analyses were performed on PerkinElmer Clarus 680/600S. MS spectra were recorded on Maldi SYNAPT G2-S HDMS (Waters) spectrometer. UV–Vis spectra were recorded using Evolution220 spectrophotometer from Thermo Scientific. XPS spectra were recorded on PHI 5000 VersaProbe X-ray photoelectron spectrometer using an Al KR X-ray source. FT-IR spectra were recorded on Jasco 6200 instrument. SEM, STEM and EDX were recorded on FEI Nova NanoSEM 450.

### General procedure for the synthesis and characterization of NP dispersions

Metal precursor(s) (0.01 mmol) and oleylamine (3 mmol, 1 mL) were loaded into 10 mL Schlenk tube. The tube was evacuated and filled with argon three times. If necessary, the degassed solvent was added. The tube was immersed into a pre-heated oil bath, and the suspension was stirred with 1 cm cylindrical bar at a speed of 1400 rpm. The samples for analyses were prepared as following. UV–Vis: aliquots of 1 % (v/v) of original volume were taken over the course of the reaction using automatic or glass pipette and diluted with chloroform; SEM, EDX and XPS: aliquots of 5 % (v/v) of original volume taken from the reaction mixture were diluted with 1:1 (v/v) EtOH-MeOH and agitated on a laboratory mixer at 240 rpm for 1 h. If necessary, the dispersion was centrifuged at 2000–3500 rpm up to 10 min followed by the decantation of supernatant. The procedure was repeated up to 5 times. The NP sediment was dissolved in chloroform and deposited on a silicon wafer or TEM grid. GC–MS, IR, NMR and MS samples were obtained from the supernatant by evaporation of solvent on rotavap.

## Results and discussion

We explored potential reductants of gold(III) oxide with a focus on substances capable of serving as ligands for AuNPs. The best results were obtained in organic media by using fatty amines. Other ligands, for example, thiols and phosphines produced polydisperse and aggregated NPs. The temperature regime was crucial to obtain high-quality NPs and secure them against aggregation. The reduction by amines required elevated temperatures (>110 °C) otherwise the reaction was sluggish furnishing NP aggregates. The NPs were also prone to aggregation at low metal–amine ratios. The NP surface was not protected enough by amine ligands leading to uncontrolled growth and sintering of NPs. The effective passivation of NP surface was only attained at the ratios above 1:80. The correlation between the chain length of amine, and the size of NPs was not as apparent as for NPs derived from gold(III) chloride (Marchetti et al. 2011). The NP sizes were in range of 6–9 nm irrespective of aliphatic amine (C12–C18) used. In turn, the alkyl chain of octylamine was too short to effectively stabilize the metal core of NPs. The reaction proceeded equally well in aromatic and aliphatic hydrocarbon solvents that is consistent with previous reports on the reduction of gold(III) chloride (Wu et al. 2013). The reaction also took place without solvent, in particular when employing oleylamine. The latter was also superior in view of uniformity of NPs formed and therefore served us as a model to study the reduction of gold(III) oxide in more detail. The obtained nanoparticles are readily redispersible in nonpolar solvents, especially in chloroform and can be further functionalized with organic thiols (1-undecanthiol, 11-mercaptoundecyl-*N*,*N*,*N*-trimethylammonium bromide).

**Fig. 1:**
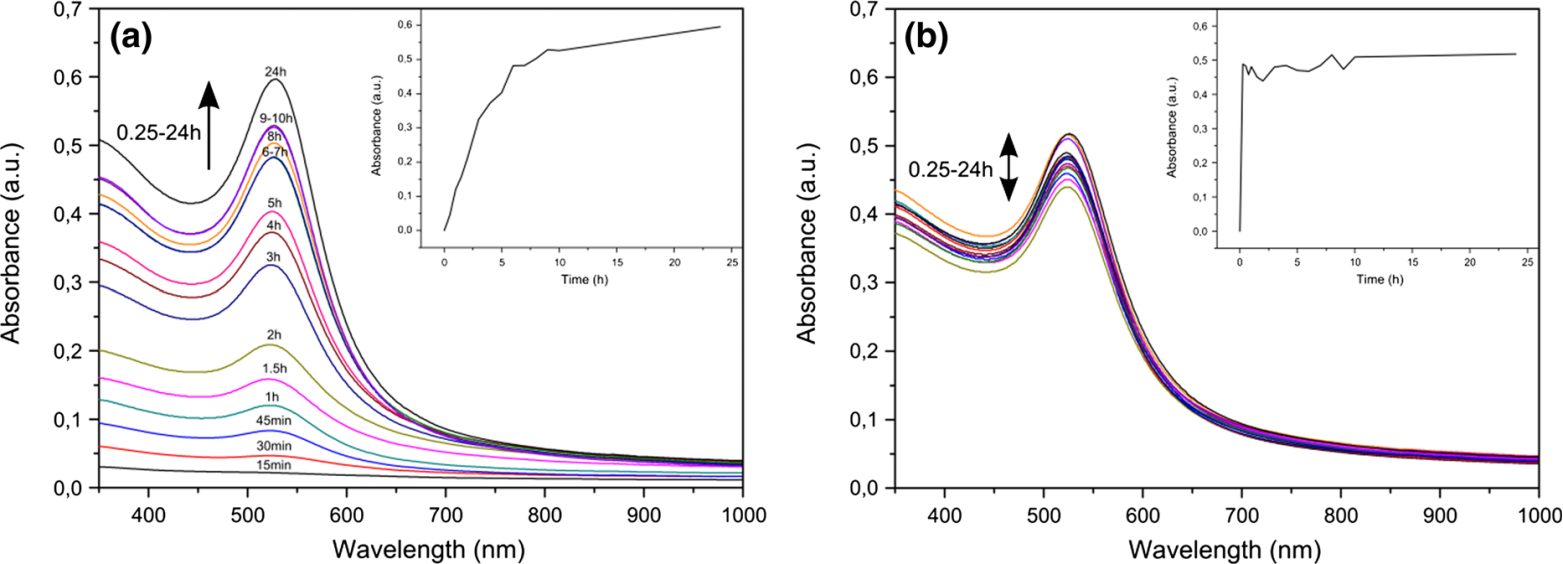
UV–Vis traces of the formation of AuNPs at 130 °C (**a**) and 180 °C (**b**). The *insets* show the absorbance as a function of time at *λ* = 526 nm

**Fig. 2:**
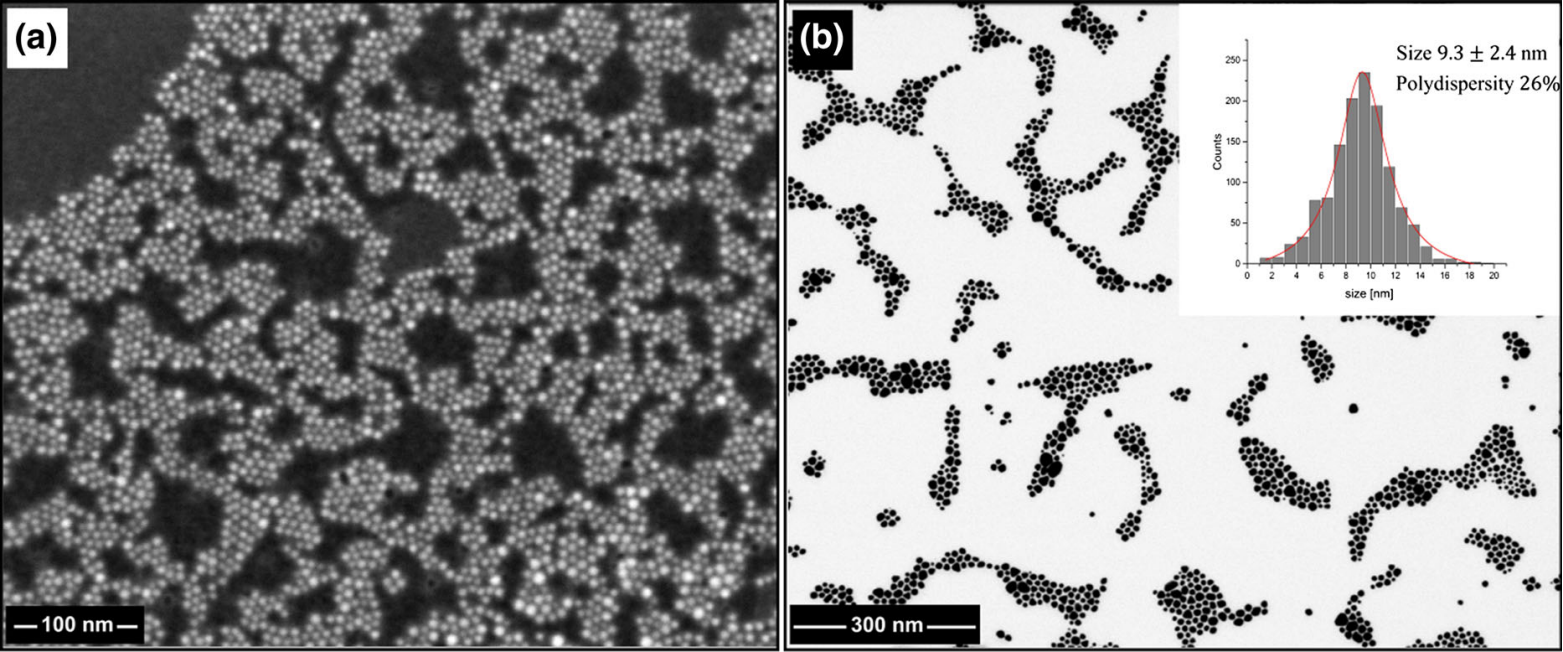
SEM (**a**) and STEM (**b**) micrographs of NPs obtained in oleylamine at 180 °C, 24 h. The *inset* shows the NP size distribution

**Fig. 3:**
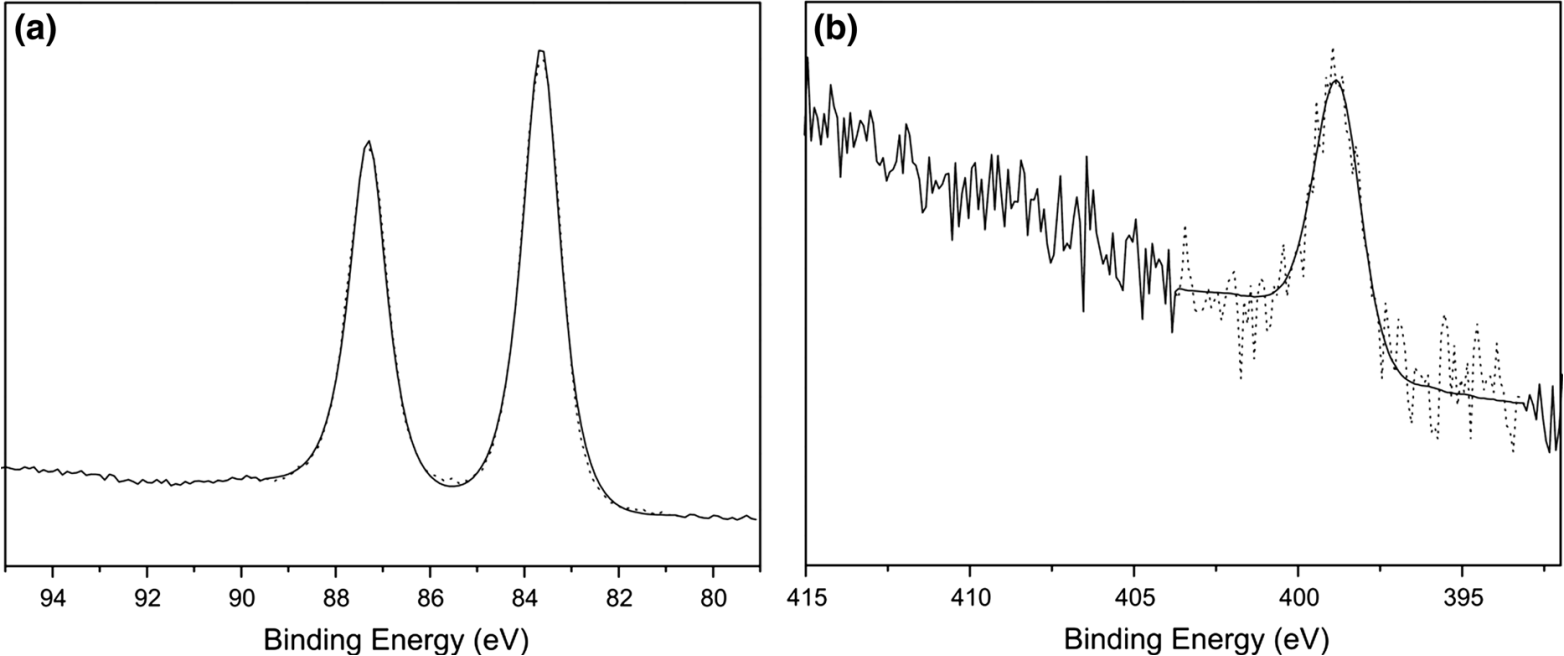
XPS evidence for the formation of amine-capped AuNPs. The experimental and fitted data are depicted as *dotted* and *solid lines*, respectively. The single components in Au4f (**a**) and N1s (**b**) regions correspond to the reduced gold (Au^0^) and non-protonated amine group (–NH_2_), respectively

In a typical experiment, the suspension of gold(III) oxide and an excess of oleylamine was vigorously stirred upon heating. The reaction was performed in neat amine (300 equiv.) under a blanket of argon. Increasing the initial amount of oleylamine (up to 1200 equiv.) had almost no influence both on the reaction time and the size of NPs. The use of protective inert gas atmosphere was necessary as the NPs were aggregated in air. During the reaction, the color of the solution gradually turned deep red indicating the formation of NPs. The appearance of surface plasmon resonance band enabled to follow the process by UV–Vis spectroscopy. The corresponding time-resolved spectra are shown in Fig. 1. For 130 °C, the plasmonic peak was increased over 24 h indicating the continuous formation of AuNPs. At higher temperature (180 °C), the reaction was virtually completed within 15 min. In both cases, the absorption maximum drifted slightly over time toward the red end of the spectrum due to NP growth. SEM imaging showed that the initially formed NPs were suffered from the broad size distribution (Fig. S1). This is not surprising taking into account the heterogeneity of the feed mixture. However, the NPs became more uniform upon prolonged heating. The best quality (in terms of dispersity) NPs were obtained at 180 °C after 24 h. The micrographs and size distribution of NPs are depicted in Fig. 2. The low polydispersity is also nicely manifested by the hexagonal arrangement of NPs as seen in low-magnification SEM images (Fig. S2). The XPS analysis revealed that the core of NPs consists of metallic gold. The binding energy for Au4f_7/2_ band (84.4 eV) is in good agreement with literature data for Au^0^ (Casaletto et al. 2006). No signals corresponding to oxidized gold species were observed indicating the quantitative formation of gold nanoparticles. The surface of NPs is covered by neutral amine ligands as evidenced by the presence of a single peak at 398.8 eV (Kumar et al. 2003). XPS spectra are shown in Fig. 3.

**Fig. 4:**
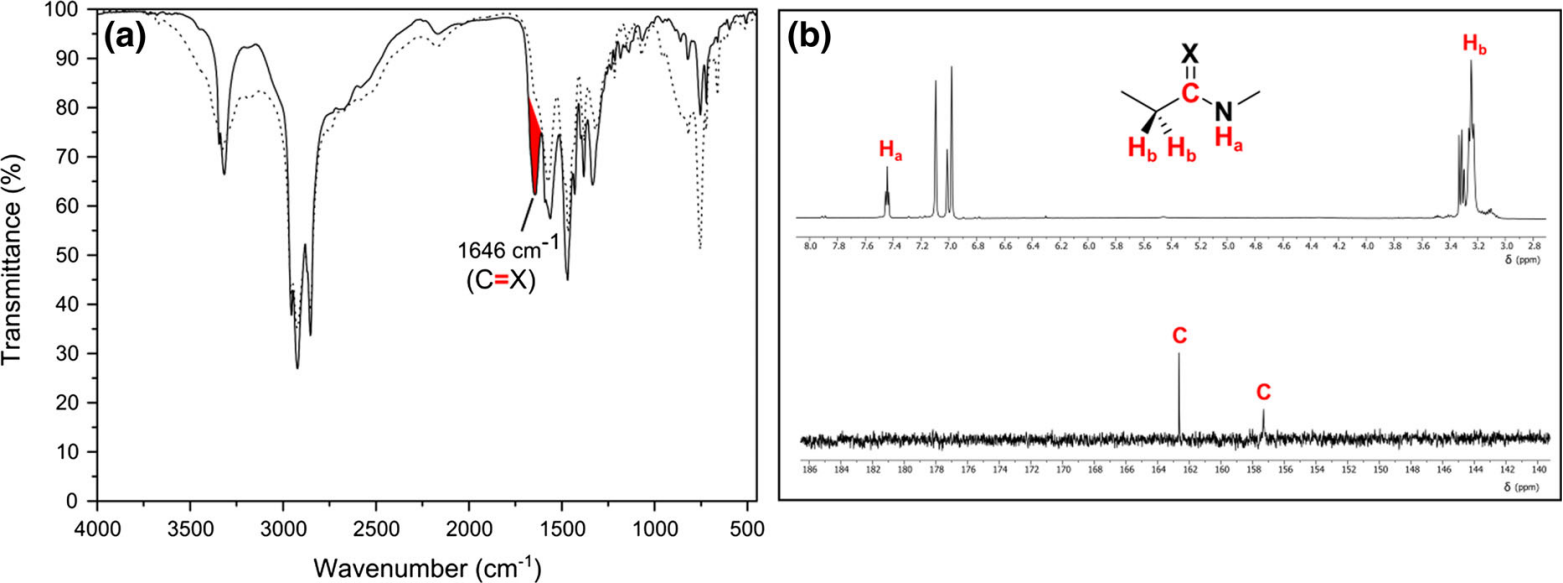
Partial IR (**a**) and NMR (**b**) spectra of postreaction mixture obtained by heating gold(III) oxide with octylamine. The reaction was performed at 110 °C in toluene-d_8_ for 24 h. IR spectrum of pure octylamine is represented as a *dotted line*. The signals characteristic for amide and amidine group are marked in *red*. (Color figure online)

**Fig. 5:**
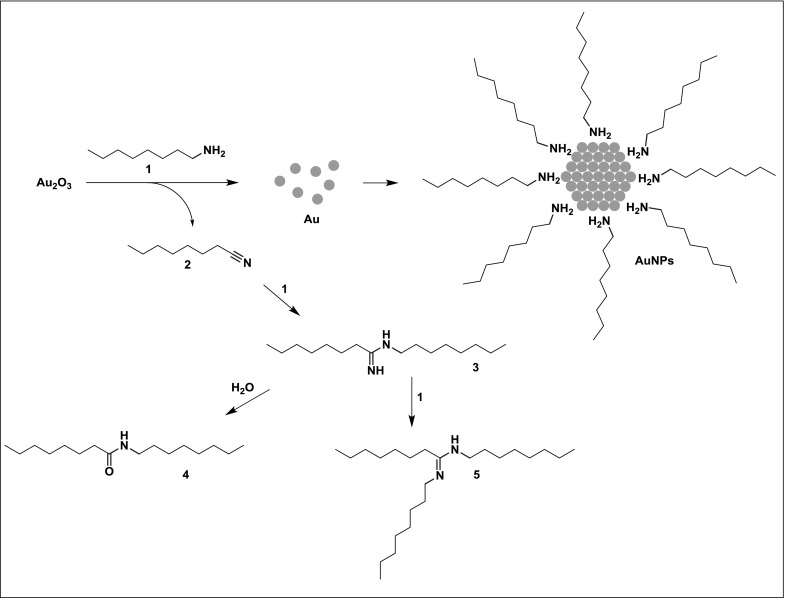
Cartoon representation of chemical processes that take place during the NP formation. The amine **1** reduces gold(III) oxide to gold and stabilizes the surface of AuNPs. The nitrile **2** which is the product of amine oxidation reacts with the amine **1** to give amidine derivative **3**. The adduct **3** undergoes next hydrolysis and aminolysis to afford **4** and **5**, respectively

It should be noted that gold(III) oxide is thermally unstable compound which spontaneously decomposes to gold and oxygen. To define unambiguously the function of amine in the process of NP formation, the gold precursor was heated without amine additive using chlorobenzene as a solvent. The experiment was conducted at 130 °C for 6 h. Neither the formation of NPs nor changes in the morphology of gold(III) oxide was noticed by SEM (Fig. S3). Nonetheless, the loss of oxygen from the sample was revealed by EDX. The rate of the spontaneous deoxygenation of gold(III) oxide, however, was much slower than that in the presence of amine. That is, the amine serves not only as a ligand but also as a reducing agent in the process. The reduction probably occurs through an electron transfer to gold ions that lead to the oxidation of amine to nitrile (Chen et al. 2007; Liu et al. 2007). For the sake of simplicity, octylamine was employed in mechanistic studies. Indeed, the presence of trace amounts of octanenitrile was detected by GC–MS (Fig. S4). Besides, the more abundant signals belonging to unidentified compounds with higher mass and amide like profile were observed. A new band at 1646 cm^−1^ that likely corresponds to stretching mode of either C=O or C=N bond was found in FT-IR spectrum (Fig. 4a). The peaks characteristic for amide and amidine moieties was also observed in ^1^H NMR spectrum (Fig. 4b). Further inspection by ^13^C NMR spectroscopy confirmed the presence of two different compounds containing C=X bond (where X is O or N). ESI–MS analysis (Fig. S5–S6) ascribed eventually the observed NMR shifts to *N*-octyloctanamide (**4**) and *N*,*N’*-dioctyloctanimidamide (**5**). The products are derivatives of *N*-octyloctanimidamide (**3**) which is formed in the reaction of nitrile (**2**) with an excess of amine (**1**) present in the solution. The fate of amine during the preparation of AuNPs is presented in Fig. 5.

**Fig. 6:**
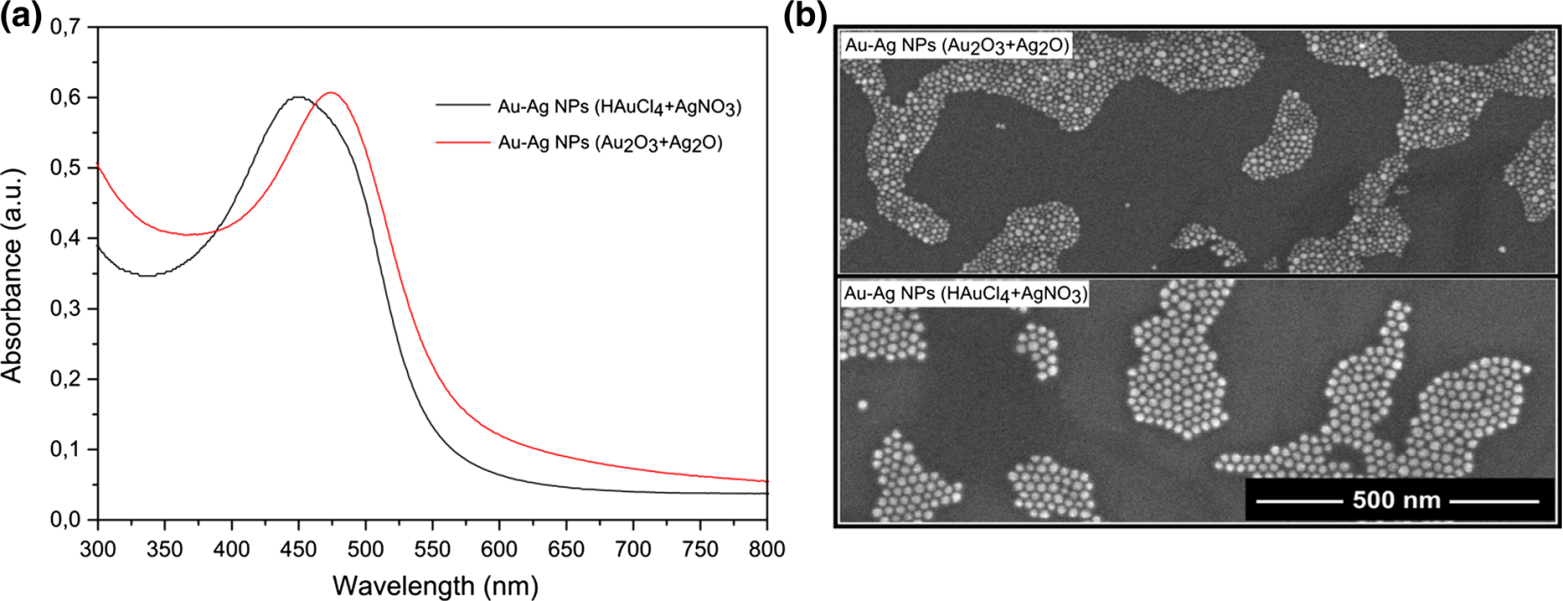
UV–Vis (**a**) and SEM (**b**) traces of Au–Ag NPs obtained in the presence and absence of chloride ions. In both cases, the reaction was performed with the same concentrations of Au and Ag precursors (0.01 M) in bulk amine at 180 °C for 3 h

The method of NP preparation is not only limited to gold but can also be extended to other metals. For example, the reduction of silver(I) oxide to silver nanoparticles took place under conditions outlined above (Fig. S7). This fact prompted us to investigate the influence of chloride ions on the formation of NPs in binary Au–Ag mixtures (Fig. 6). A trend toward decreasing the size of NPs was also maintained in this case. The NPs obtained by co-reduction of gold and silver oxides were significantly smaller than those obtained from chloroauric acid and silver nitrate (Fig. 6b). The difference was also observed in the composition of the NPs. The NPs obtained in the presence of chloride ions had a lowered content of silver due to the precipitation of silver chloride (Fig. S8). Furthermore, the structure of these NPs seems to be inhomogeneous with a core and a shell enriched in gold and silver, respectively. This is indicated by the shift of plasmonic band toward shorter wavelength despite the precipitation of AgCl (Fig. 6a). There is also an apparent shoulder near 500 nm that might be diagnostic of the separation of metal phases (Lu et al. 2013; Zeng et al. 2014). The generation of gold seeds was observed visually by the appearance of red color shortly after the reaction was started. The color next rapidly changed through brown (reduction of Ag^+^) to dark (sedimentation of the NPs). The observed reduction sequence has been described previously (Wang et al. 2009). Conversely, the NPs obtained from metal oxides appears to be efficiently alloyed retaining 1:1 Au–Ag feed ratio as evidenced by plasmonic peak centered at 473 nm (Rajendra et al. 2015).

## Conclusions

In summary, we developed a new method for obtaining noble metal NPs from corresponding oxides by reducing with aliphatic amines. The NPs obtained by this method are usually smaller than those prepared using halogen-containing metal precursors. For example, the reduction of gold(III) oxide yields sub-10 nm particles with good monodispersity. The lack of halogen has also influence on the final composition of NPs. The NPs made of gold and silver are alloyed better than those obtained in the presence of halogen. Finally, and importantly, the presented approach adheres well to green chemistry principles. The synthesis is performed in the absence of organic solvents and the reagents used are non-toxic (gold(III) oxide) and manufactured from natural oils (oleylamine).

## Electronic supplementary material

Below is the link to the electronic supplementary material.

Supplementary material 1 (DOCX 4027 kb)
