# Supplementary material for: A halogen-free synthesis of gold nanoparticles using gold(III) oxide
Source: J Nanopart Res. 2016 Aug 29;18(9):261. doi: 10.1007/s11051-016-3576-x (PMC5003902; doi:10.1007/s11051-016-3576-x)
Supplement: Supplementary file 1 — Supplementary material 1 (DOCX 4027 kb) [file 11051_2016_3576_MOESM1_ESM.docx]

**A halogen-free synthesis of gold nanoparticles using gold(III) oxide**

Volodymyr Sashuk* and Konrad Rogaczewski

*Institute of Physical Chemistry, Polish Academy of Sciences, Kasprzaka 44/52, 01-224, Warsaw, Poland*

[*vsashuk@ichf.edu.pl*](mailto:vsashuk@ichf.edu.pl)

**Supporting Information**

# Spectroscopy and microscopy data


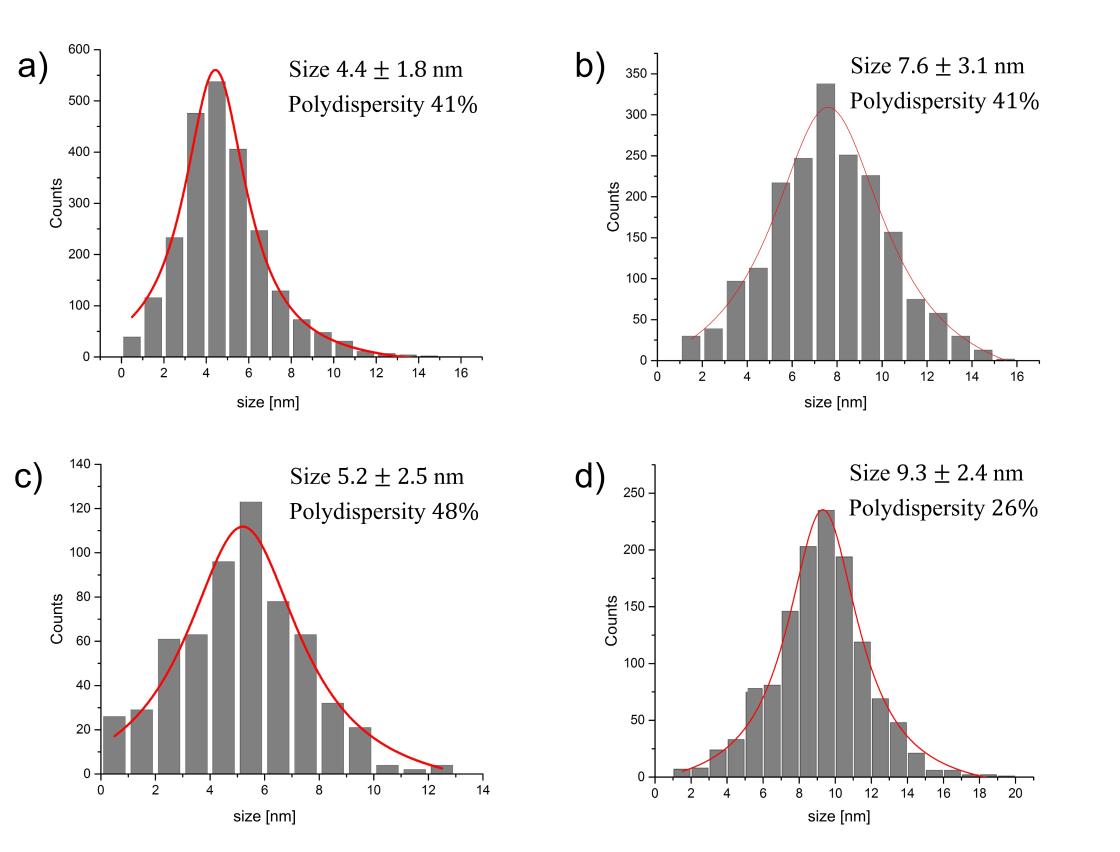


Fig. S1. The dependence of the size of AuNPs on the temperature and the reaction time: (a) 130°C, 3h; (b) 130°C, 24h; (c) 180°C, 3h; (d) 180°C, 24h. The reaction was performed in oleylamine.


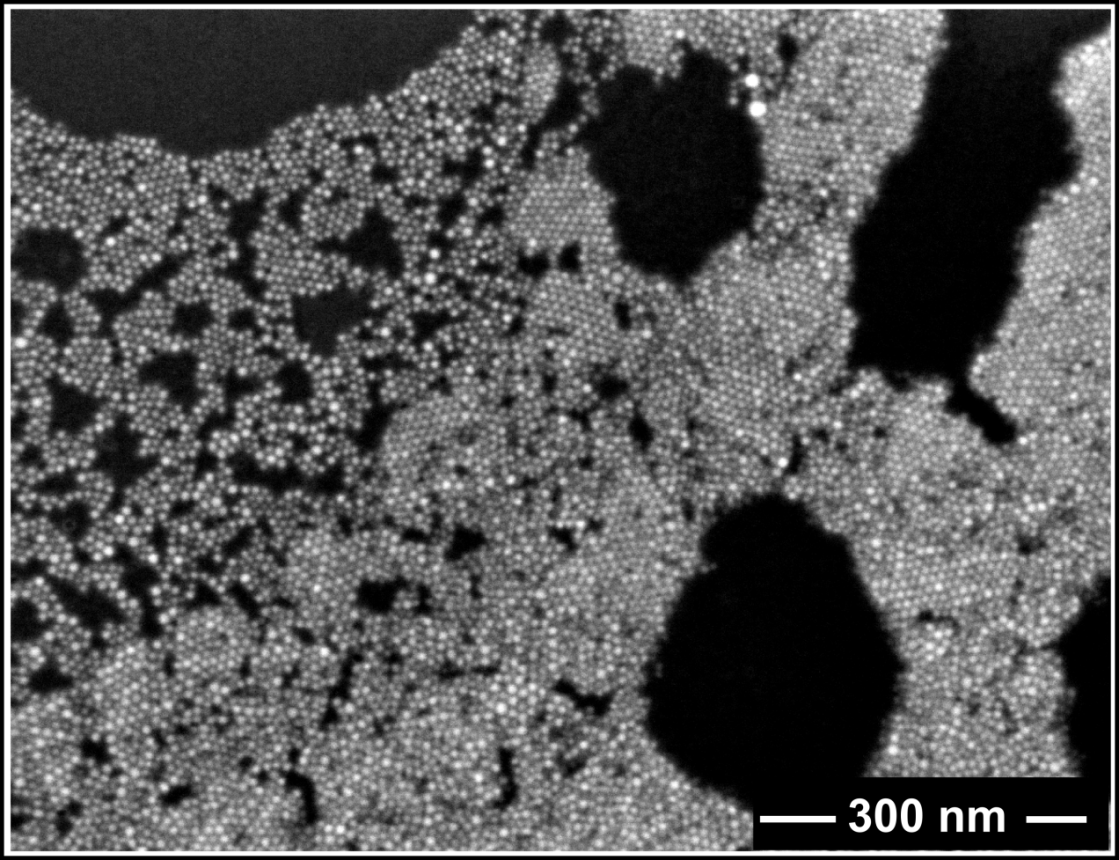


Fig. S2. SEM micrograph of AuNPs, oleylamine, 180°C, 24h.


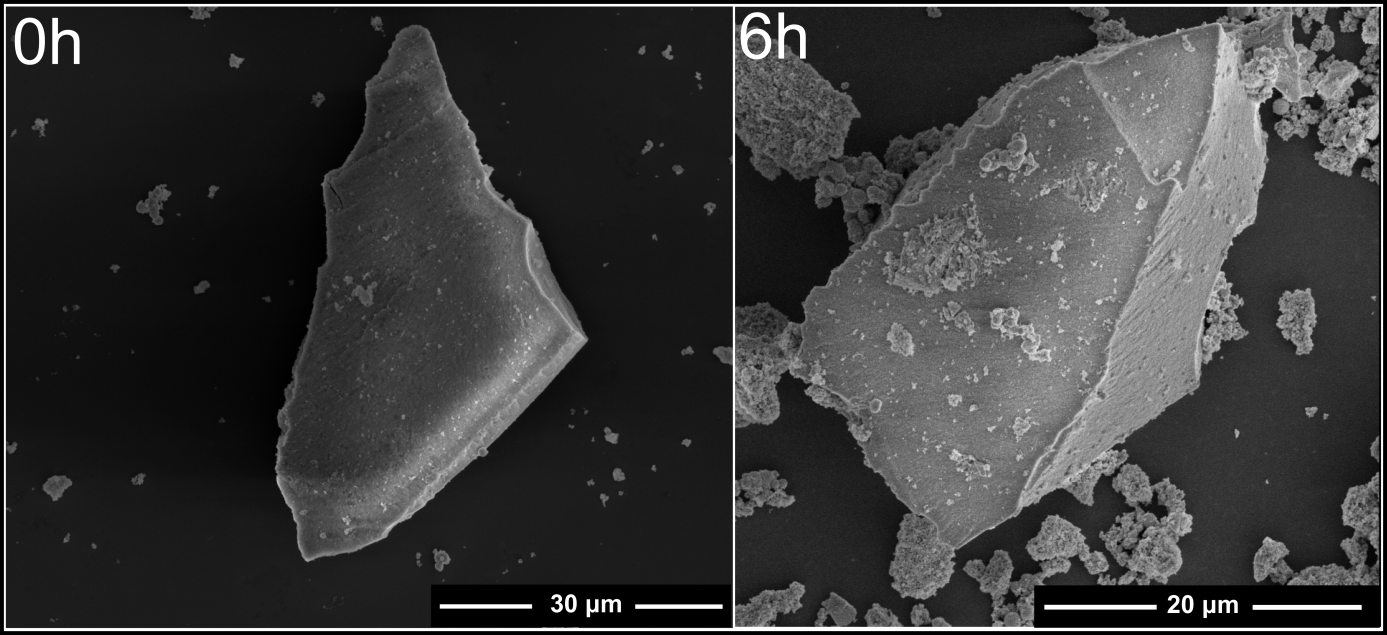


Fig. S3. SEM micrographs of Au_2_O_3_ lamps before (left side) and after heating (right side) in chlorobenzene at 130°C for 6h. According to EDX the ratio of Au:O changed from 41:59 to 76:24.


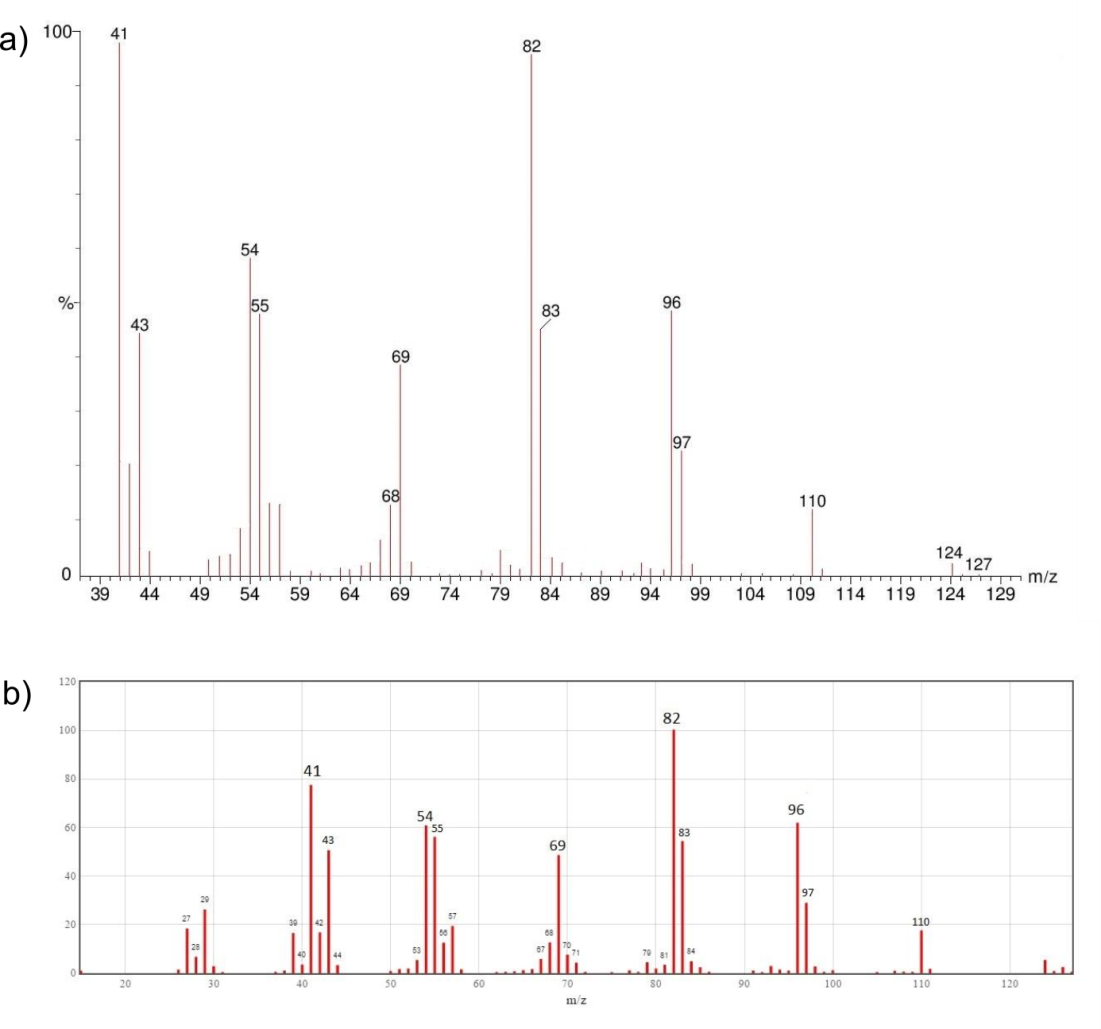


Fig. S4. MS profile of octanenitrile: (a) experimental GC-MS data; (b) NIST database.


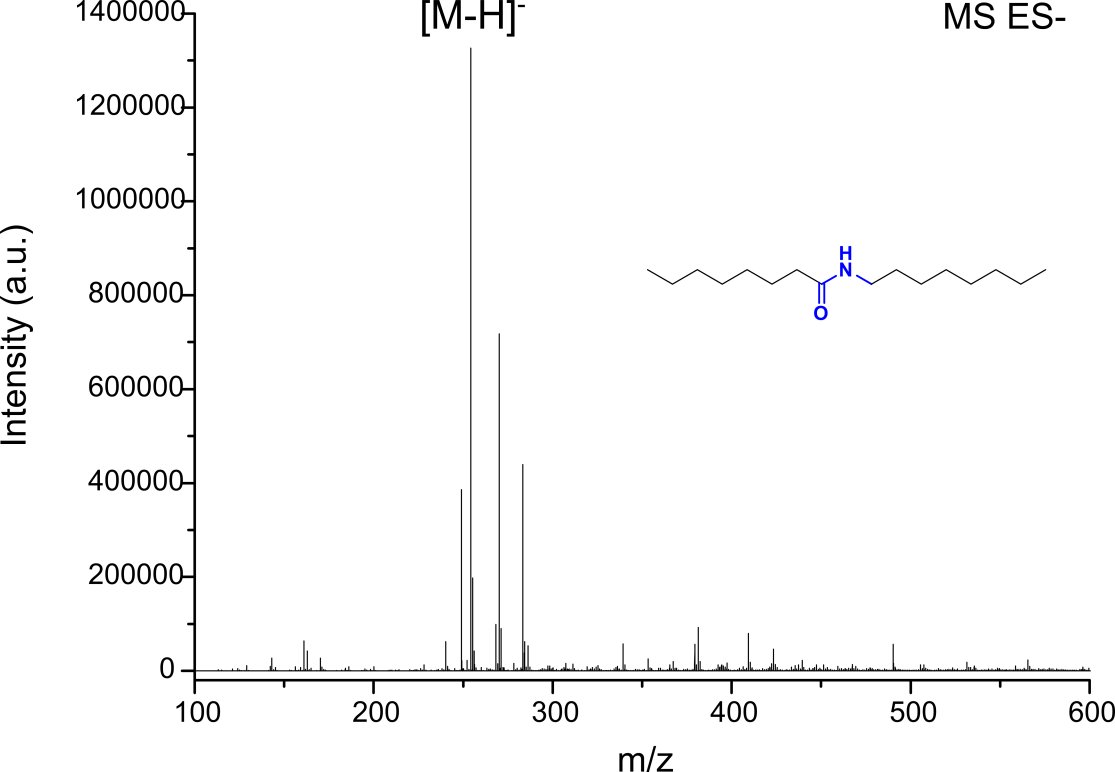


Fig. S5. MS spectrum of postreaction mixture from the reaction between gold(III) oxide and octylamine, toluene-d_8_, 110°C, 24h (negative ion mode).


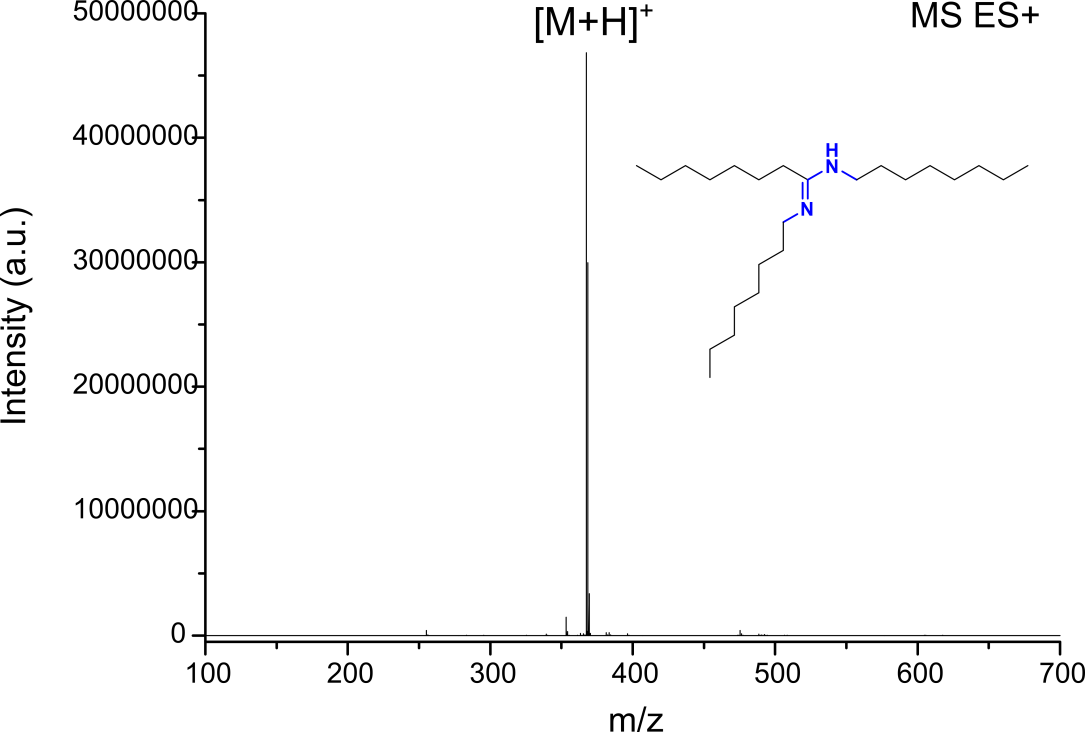


Fig. S6. MS spectrum of postreaction mixture from the reaction between gold(III) oxide and octylamine, toluene-d_8_, 110°C, 24h (positive ion mode).


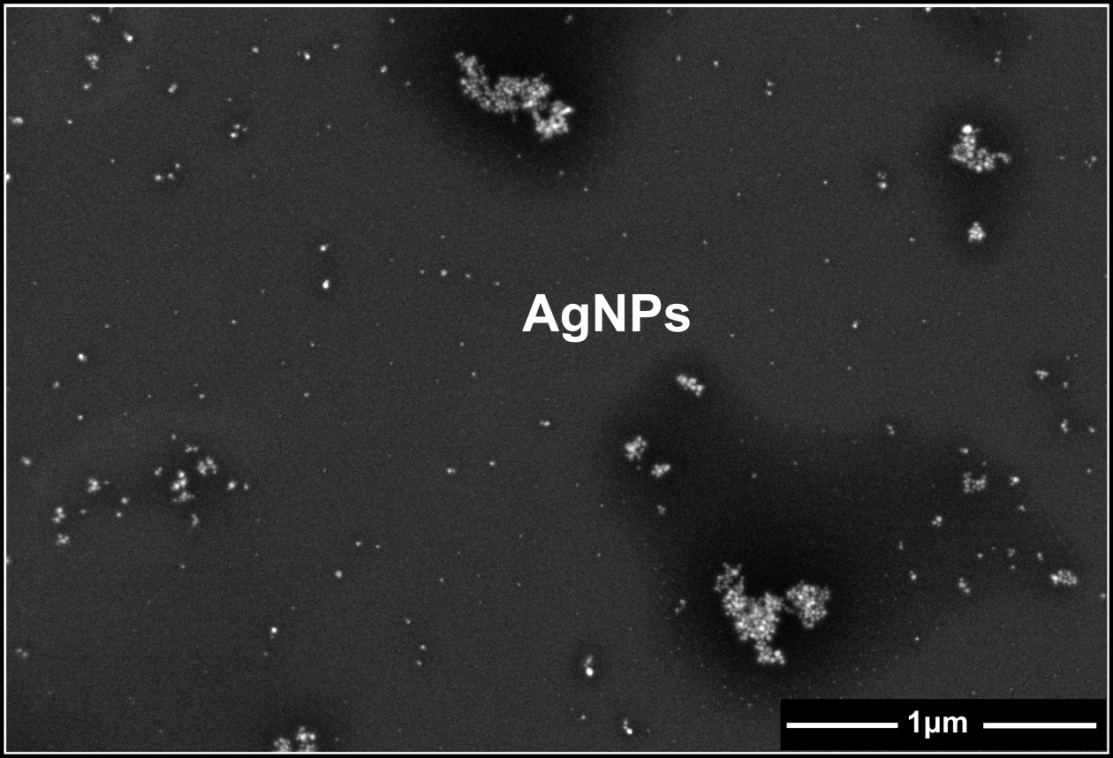


Fig. S7. SEM micrograph of AgNPs, oleylamine, 130°C, 3h.


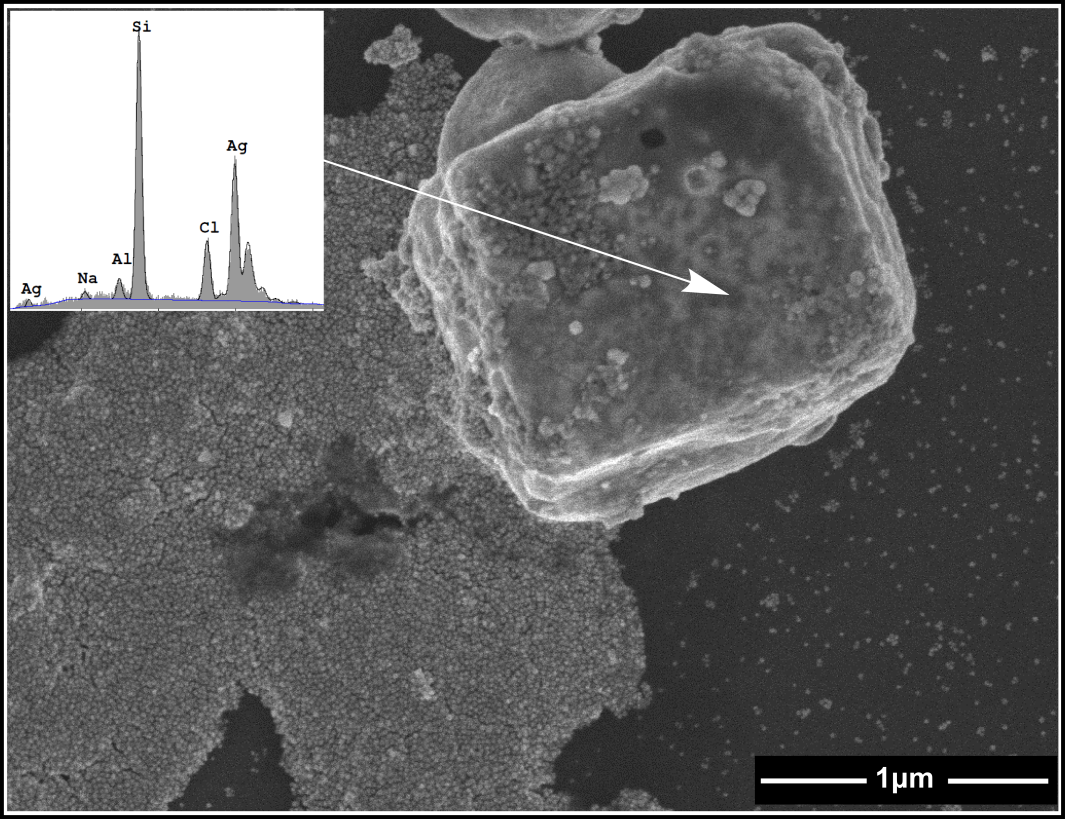


Fig. S8. SEM micrograph of postreaction mixture from the reaction between chloroauric acid and silver nitrate in oleylamine, 180°C, 3h. The inset shows the atomic composition (EDX) of the nanocube.
